# Supplementary material for: Protocol for black student well-being study: a multi-site qualitative study on the mental health and well-being experiences of black UK university students
Source: BMJ Open. 2022 Feb 9;12(2):e051818. doi: 10.1136/bmjopen-2021-051818 (PMC8830255; doi:10.1136/bmjopen-2021-051818)
Supplement: Author's manuscript [file bmjopen-2021-051818.draft_revisions.pdf]

BMJ Open

BMJ Open is committed to open peer review. As part of this commitment we make the peer review history of every article we publish publicly available.

When an article is published we post the peer reviewers' comments and the authors' responses online. We also post the versions of the paper that were used during peer review. These are the versions that the peer review comments apply to.

The versions of the paper that follow are the versions that were submitted during the peer review process. They are not the versions of record or the final published versions. They should not be cited or distributed as the published version of this manuscript.

BMJ Open is an open access journal and the full, final, typeset and author-corrected version of record of the manuscript is available on our site with no access controls, subscription charges or pay-per-view fees (http://bmjopen.bmj.com).

If you have any questions on BMJ Open's open peer review process please email info.bmjopen@bmj.com

BMJ Open

Protocol for Black Student Wellbeing Study: A multi-site qualitative study on the mental health and wellbeing experiences of Black UK university students

Journal:	*BMJ Open*
Manuscript ID	bmjopen-2021-051818
Article Type:	Protocol
Date Submitted by the Author:	30-Mar-2021
Complete List of Authors:	Stoll, Nkasi; Institute of Psychiatry Psychology and Neuroscience Division of Academic Psychiatry, Psychological medicine; King's College London, Centre For Society and Mental Health<br>Yalipende, Yannick; University College London, Division of Psychology and Language Sciences<br>Arday, Jason; University of Durham Department of Sociology and Social Policy, Higher Education and Social Inequality<br>Smithies , Dominic; Student Minds<br>Byrom, Nicola; King's College London, Department of Psychology<br>Lempp, Heidi; King's College London, Inflammation Biology<br>HATCH, STEPHANI; Institute of Psychiatry Psychology and Neuroscience Division of Academic Psychiatry, Psychological medicine; King's College London, Centre For Society and Mental Health
Keywords:	MENTAL HEALTH, QUALITATIVE RESEARCH, PSYCHIATRY, Health policy < HEALTH SERVICES ADMINISTRATION & MANAGEMENT, EDUCATION & TRAINING (see Medical Education & Training)

SCHOLARONE™
Manuscripts

Protocol for Black Student Wellbeing Study: A multi-site qualitative study on the mental health and wellbeing experiences of Black UK university students

Ms Nkasi Stoll[1,2] Mr Yannick Yalipende[3], Dr Jason Arday[4], Mr Dominic Smithies[5], Dr Nicola Byrom[6], Dr Heidi Lempp[7]*, Professor Stephani Hatch[1,2]*

[1]King's College London, Institute of Psychiatry Psychology and Neuroscience, Department of Psychological Medicine, London, UK.

[2]King's College London, ESRC Centre for Society and Mental Health, London, UK.

[3]University College London, Division of Psychology and Language Sciences, London, UK.

[4]Durham University, Department of Sociology, Higher Education and Social Inequality, UK.

[5]Student Minds, the UK's student mental health charity, 17 Springfield Mount, Woodhouse, Leeds LS2 9NG.

[6]King's College London, Institute of Psychiatry Psychology and Neuroscience, Department of Psychology, London, UK.

[7]King's College London; Faculty of Life Sciences and Medicine, Department of Inflammation Biology, Centre for Rheumatic Diseases, London UK.

Corresponding Author:

Ms Nkasi Stoll, BSc, MSc

King's College London, Institute of Psychiatry Psychology and Neuroscience

Department of Psychological Medicine

16 De Crespigny Park

London, SE5 8AF, UK

Email: Nkasi.1.stoll@kcl.ac.uk

Twitter: @NkasiStoll

For peer review only

Abstract

Introduction: There is increasing concern about the mental health and mental wellbeing of university students in the United Kingdom (UK). Black university students who report a mental health condition are less likely to complete their course, achieve a first-class or upper second-class degree, and progress to further education. This study will document Black university students' accounts of their mental health experiences and perceptions of key turning points of biographical changes to their mental health as they move through the university life cycle.

Methods and Analysis: This is a qualitative study. Data will be collected through a Biographical Narrative Interpretive Method. Interviews will enable the researcher to study systematically how participants make sense of themselves and account for the complexities of their life experiences, from their own perspectives and language. An interpretative Phenomenological Approach will be used to offer insights into what Black students studying at UK universities report affects their mental health and wellbeing. Data collection for this study commenced in October 2020 and analyses will be completed by January 2022.

Ethics and Dissemination: Full ethical approval for the current study was obtained from King's College London Psychiatry, Nursing and Midwifery Research Ethics Subcommittee (Rec Ref: 20489, Project Ref: HR-19/20-20489, 2nd October 2020). From the study findings, we aim to contribute to the evidence base, make recommendations for interventions, and encourage further study into Black student mental health.

For peer review only

Strengths and Limitations of this study

- The current study is designed to be the first multi-site UK study to explore, from Black undergraduate and postgraduate university students' perspectives, the life events and experiences that effect their mental health and wellbeing throughout the university life-cycle.
- A multi-disciplinary coding team (including Black university students) will ensure the inclusion of multiple perspectives in order to reduce researcher bias and provide opportunities to discuss disagreements in interpretations.

For peer review only - http://bmjopen.bmj.com/site/about/guidelines.xhtml

- This study will contribute to the evidence base on race, mental health and higher education; provide relevant recommendations for interventions; and encourage further study into Black student mental health.
- The current project is exploring the lives of only 20-25 Black students studying at 10 UK universities, so may lack generalizability, representativeness and transferability of findings.

Introduction

The mental health and mental wellbeing of university students in the United Kingdom (UK) is of increasing concern to policymakers, researchers, charities, university staff and students[1-4]. Young adulthood is a critical age for emotional, psychosocial and cognitive development[5-6] and first onset of mental health problems, suicidality, and substance misuse problems[7-9]. Young adulthood is also the time that many enter and navigate university in the UK. There are approximately 2.4 million university students in the UK, the majority being young adults[10], which means that Higher Education Institutes (HEI) are in the best position to provide prevention and intervention to many young adults during an important transitional period.

Throughout the university life-cycle[11], students are exposed to a range of experiences that make higher education a high-risk period for maladaptive coping and possible onset of mental health problems, including (but not limited to): individuation, separation from family, new social connections, increased autonomy and responsibility, academic-related stress, financial concerns, sleep disruption, balancing conflicting demands of studying with personal and family life, and exposure to risky behaviours, including recreational drug use and alcohol binging[12-16]. Unsupported mental health problems are associated with progression to other co-morbid disorders, substance use disorders, self-harm and suicide ideation and attempts[17-20].

A growing body of evidence explores inequalities and inequity in student admissions, experiences and outcomes in UK higher education by race, gender, class and (dis)ability[21-27]. Black students with a mental health problem studying at UK HEI are less likely to complete their course, achieve a first- or upper second- class degree, and progress to further education[28-29]. The Office for Students[2] reported that "black students with mental health conditions are being failed throughout the student cycle" (pg. 6), but that there is limited data or research available to explain how and why. Available research indicates the following factors and dimensions may explain the inequality and inequity observed in higher education, at the

For peer review only - http://bmjopen.bmj.com/site/about/guidelines.xhtml

expense of Black students: racism and structural racism, discrimination, mental health stigma, sexism, cultural incompetence and insensitivity of healthcare professionals[30-32]. It is essential to know more about the experiences of, risk factors for, and challenges that affect Black university students' mental health in UK higher education in order to (re-)design, develop and deliver appropriate mental health and wellbeing services.

Critical Race Theory

Critical race theory (CRT) offers an interpretive theoretical approach to explore and challenge racial inequality in society. It is based on the understanding that racism is embedded as normal practice within society and institutions, rooted in slavery and colonialism; and race is a socially constructed concept that is used by White people to further their socio-economic and political interests and power at the expense of people of colour. This racial bias causes discrimination within law, employment, housing, healthcare, politics, and education, disadvantaging people of colour[33-34]. CRT in the educational context explores and challenges the White supremacist patriarchal structures and assumptions that have historically shaped education[33,35]. CRT theorists explore racial inequality in: admissions, curriculum and pedagogy, teaching and learning, institutional culture, campus racial climate, and policy and finance within UK further and higher education[36-40]. CRT is also used as a conceptual lens through which to interrogate the eurocentric, ethnocentric and egocentric ways in which psychological research and mental health practices disadvantage people of colour[41-42]. Therefore, CRT will guide the direction of this current study by providing a framework to support the study team to interrogate the methodology, analyses, and interpretation throughout the research process[32].

Aims

The aim of this study is to document and explore Black university students' (i) accounts of their mental health experiences as they transition throughout their university lives; and (ii) perceptions of key turning points of biographical change to their mental health in their lives as they move through the university student life cycle.

Objectives

This study will use Biographic Narrative Interpretive Method (BNIM)[43-44] interviews and Interpretative Phenomenological Approach (IPA)[45-52] to get an in-depth understanding of the educational and mental health of experiences Black UK university students.

For peer review only - http://bmjopen.bmj.com/site/about/guidelines.xhtml

Research questions

1. From the students' perspective, what life events and experiences do Black university students perceive affect their mental health and wellbeing?
2. How does institutional and structural racism within UK education systems affect the mental health and wellbeing experiences and outcomes of Black university students?

Methods and Analysis

Study design

This is a qualitative study. Data is being collected through BNIM interviews[43-44]. This interview technique enables the researcher to study how participants make sense of themselves and account for the complexities of their life experiences, from their own perspectives and choice of language[43-44]. For the current study, IPA[45-52] offers insights into what Black students studying at UK universities report affects their mental health and wellbeing. Data collection for this study commenced in October 2019 and analyses will be completed by January 2022.

Recruitment

A webpage has been set up specifically for the study on the Health Inequalities Research Network (HERON) website: www.heronnetwork.com which is General Data Protection Regulation (GDPR) complaint. The webpage has the lead researcher (N.S.)'s contact information, the study's rationale, expression of interest form, information sheet, consent form, list of support services, updates about the timeline of the project, and any publications of findings. The study is advertised on social media accounts (Twitter and Instagram) set up specifically for the study. Social media posts with a short summary of the study have been designed and sent out on this social media page to target participants. The social media posts include the study's website and QR code which means that potential participants can access the webpage with one click. Recruitment emails have been sent to student unions, student groups and student services in the UK.

Completed expression of interest forms are automatically sent to a KCL study mailbox which has been set up for the study. N.S. monitors the incoming forms twice a day and screens the

For peer review only - http://bmjopen.bmj.com/site/about/guidelines.xhtml

participants based on the inclusion and exclusion criteria. Potential participants are contacted by N.S. via email to complete an attached consent form which they are asked to email back to the researcher. Participants have a minimum of 48 hours to decide whether they would like to participate in the study. If the participant has not responded after two days they are sent a reminder email by N.S. and subsequent two reminder emails until they are considered non-responsive.

Sample size

Purposive sampling[51] is being used to recruit Black university students who meet the inclusion criteria. 15-20 students are being recruited from nine Russell and non-Russell group universities across the UK. Data from the Higher Education Statistics Agency (HESA)[10] for UK higher education student enrolments by higher education provider and ethnicity for the academic years 2014/15 to 2018/19 was used to decide which universities to recruit from. In Microsoft Excel the data was sorted into higher education providers with the highest percentage of Black students enrolled, to the lowest. After this, inter-quartile range was calculated to divide the higher education providers into quartiles of median, lower (i.e. lowest percentage of Black students) and upper range (i.e. highest percentage of Black students). 3 higher education providers that fall into each range were chosen.

Inclusion and Exclusion criteria

The inclusion criteria for students are deliberately broad to include students who: (1) self-identify as Black (Caribbean, African, and mixed heritage with Black); (2) are currently studying at, within a year of graduating from, or are within a year of dropping out of a university course; (3) self-identify as having struggled with their mental health and/or wellbeing whilst studying at a UK university; (4) are aged 18 or over. As an IPA study[45-52], this will allow for a wide range of Black university students, from different backgrounds, to be eligible for study inclusion as the study processes.

The exclusion criteria are people who: (1) have never been enrolled on a course at a UK university, (2) are enrolled at a university outside of the UK, (3) do not identify as Black (Caribbean, African, and mixed heritage with Black), (4) graduated or dropped out of university over a year ago, (5) are aged 17 or younger.

For peer review only - http://bmjopen.bmj.com/site/about/guidelines.xhtml

Potential participants are asked to complete an expression of interest form online, with sociodemographic information to determine their eligibility for the study.

Data collection

All interviews are being conducted by N.S. In order to generate a narration that has not been structured by the study team's research questions, but by the student's structure of relevance, students are invited to participate in an adapted version of (BNIM)[43-44,53]. This approach to data gathering is an open narrative interview process. The interviewer is encouraged to participate in active listening, where the interviewer does not interrupt with questions and allows the interviewee to begin, construct and end their narrative on their own terms.

The BNIM interviewing process begins with the first session where the students are asked a single question called a 'Single Question Inducing Narrative' (SQUIN)[43-44] to describe their life, in terms of their mental health and wellbeing. The SQUIN reads as follows: *"As you know, I am researching mental health and wellbeing experiences of Black university students in the UK. I understand that you have had such experiences. So please can you tell me the story of your mental health experiences before university and whilst studying at university. Please include all the events and experiences that were important for you, personally, up until now. There's no rush, you can start wherever you like. We've got 2 hours in total. I'll listen first, I won't interrupt. I'll tell you if we are running out of time. I'll just take some notes and when you've finished telling me about it all we will have a 15-minute break. After the break I may ask you further questions about your story"*. As the student is talking, N.S. takes notes on what Wengraf terms 'Particular Incident Narratives' (PINs)[43-44], which are significant events that have occurred in each student's life surrounding their mental health and wellbeing using the keywords that they use. Students are then invited to take a 15-minute break and during this time N.S. identifies questions called narrative/N-pointed questions[43-44], to clarify or pick up on PINs[43-44]. The second session begins with N.S. asking the student to reflect back on the identified PINs and continue with the N-pointed questions. The interview ends with the question: "Before we finish, is there anything you would

For peer review only - http://bmjopen.bmj.com/site/about/guidelines.xhtml

like to add that we didn't talk about?". Interviews last up to 2 hours in total. The interviews elicit two transcripts of narrative for data analysis from each student.

Interviews are conducted virtually using Microsoft Teams and are recorded with the student's permission. N.S. transcribes the interviews by hand using Microsoft Teams' automated speech-to-text transcript programme, before analysis.

A BNIM pilot study was conducted to assess comprehension of the BNIM question, relevance, timing, and research burden[54]. Two Black University students were invited to participate in the pilot study. Data was presented to the fifth, sixth, and seventh authors of this paper to collaboratively agree whether they matched the study objectives. No amendments were made to the study.

Data collection for this study commenced in October 2020 and data collection and analyses are planned to be completed by January 2022.

Data analysis

Data analysis is due to begin June 2021. Data will be analysed using IPA[45-52] on NVivo Qualitative Data Analysis Software (version 12). An inductive analysis of each student's account will be conducted before moving onto group analysis[47]. Analysis will begin with multiple readings of each student's transcript by N.S., who will make notes on the student's language and structure (including repetitions, exaggerations, emphasis, symbols, pauses, slang), emotional responses, and any insights into the students' experience and perspective on their world i.e. 'codes'. This is to interpret what and how the student is communicating their story. As the analysis develops, N.S. will look for patterns in the codes to create emerging themes. Themes will identify recurring patterns of meaning (ideas, thoughts, feelings, topics of importance or concern) presented by the student and interpreted by N.S. throughout the text. Themes will eventually be linked and grouped together to create subordinate themes. The final set of themes will be summarised, arranged into clusters to form master themes and placed into a table. Quotations from the students will be selected to illustrate each theme. All the transcripts will be collated and re-examined by N.S. who will pay attention to thematic overlap and differences in student's accounts and will make links to existing psychological and sociological

For peer review only - http://bmjopen.bmj.com/site/about/guidelines.xhtml

literature, concepts and theories which provide an in-depth understanding of the phenomena and strengthen the robustness of the data analysis. The authors will be careful to apply a critical race lens on the existing theoretical knowledge within this area of research to explain the phenomena[55].

In IPA "the participants are trying to make sense of their world; the researcher is trying to make sense of the participants trying to make sense of their world" (pg. 53[56]). The dialogue between the researcher and the participant is described as a double hermeneutic and IPA recognises the importance of constant self-awareness and reflexivity[57]. N.S. will write notes in a reflective diary throughout study conception to dissemination to draw their observations and reflections about the interview experience, data analysis and interpretations; or any other thoughts and comments of potential significance[57-59].

A second coder, a Black university student (Y.Y.), will code 20% of the interviews using IPA for cross-validation of the themes[60-61]. The authors of this paper will form a multi-disciplinary coding team and participate in conversation circles over Microsoft Teams to validate the data, participate in theme development, and promote reflexivity and dialogue[60,62]. The authors all have extensive knowledge and experience on student mental health and mental health inequality in the UK. The circle mediator (N.S.) will participate in the conversation to present and clarify ideas or issues, and encourage the dialogue. The conversation circle will be audio recorded with verbal consent of the attendees. During the conversation the objectives, methodological process, and research results will be presented. The authors will be encouraged, in a dialogic process, to give their opinion about the codes and emergent themes. A second conversation circle will be arranged for the subordinate themes.

Data protection

All confidential data will be kept for 10 years according to KCL Records and Data Retention Schedule (2019)[63]. Data will be stored on password-protected computers in password protected, encrypted folders on KCL SharePoint which will only be identifiable by the code number and only accessible to members of the research team. During transcription, audio-recordings will be anonymised, with all identifiable information removed prior to using NVivo Software. All audio-recordings will be destroyed immediately after transcription. This is

For peer review only - http://bmjopen.bmj.com/site/about/guidelines.xhtml

important to ensure that their identities are protected and they are not at risk of harm due to damage of their reputation or social standing for example, should they express controversial or negative opinions or experiences. This is particularly relevant considering the sensitive topic of study and the fact that participants are invited to consider current as well as historical experiences at university.

Ethics and Dissemination

Full ethical approval has been granted by KCL Psychiatry, Nursing and Midwifery Research Ethics Subcommittee (Rec Ref: 20489, Project Ref: HR-19/20-20489, 2nd October 2020). Written informed consent to participate and audio-recorded interviews is obtained from all participants. Participants consent to the inclusion of material pertaining to themselves, that they acknowledge they cannot be identified via the manuscript; and that the participant has been fully anonymised by the author. Data management and storage is subject to the UK Data Protection Act 1998 and GDPR. The authors assert that all procedures contributing to this work comply with the ethical standards of the relevant national and institutional committees on human experimentation and with the Helsinki Declaration of 1975, as revised in 2008.

Safety Considerations

Potential risks of participating come as a result of the participant disclosing a historical or current distressing experience. During the consent process and at the beginning of the interview, the interviewer (N.S.) informs each participant that confidentiality needs to be broken if they disclose personal details which raises concerns to N.S. about the participant's safety, the safety of others, safety of someone under the age of 18 or about a previously non-disclosed serious crime they or someone else has committed or is about to commit.

When a participant discloses a serious immediate risk the interview is stopped, a conversation is had with the participant (where appropriate) to ensure they understand why and how information is shared for their safety, and the emergency services is contacted immediately. The co-senior author (S.H.) is informed immediately after (in person, or by phone if this is not possible). Incidents are reported to the Chair of the Ethics Committee. When a participant becomes distressed during the interview N.S. asks the participant if they would like a break. When the participant would like to terminate the interview or N.S. decides the participant is no longer able to continue she offers to re-schedule the interview at the next convenient time.

For peer review only - http://bmjopen.bmj.com/site/about/guidelines.xhtml

Time is explicitly allocated to a 'debrief' period at the end of the interview for all participants to reflect upon the session, ask any questions or raise anything that may be of concern. Participants are asked: "Is there anything you would like to share regarding your experience talking to me today?". A list of support services available across the UK are listed in a document created for this research project, which is available on the research website in case participants need professional support. Every participant is sent an email directing them to this list of support services after the interview.

Output and Dissemination

Findings will be disseminated via an internal report (doctoral thesis), peer-reviewed journal articles (e.g., commentary piece(s), theoretical article(s), write up of the main findings) and conference presentations (e.g. Advance HE, The Student Mental Health Research Network [SMaRteN], AMOSHEE, The Office of Students [OFS]). The study team will seek funding from the London Interdisciplinary Social Science Doctoral Training Partnership (LISS-DTP), KCL, and the Centre for Society and Mental Health (CSMH) to design and deliver internal and external public engagement events to discuss the study findings and potential recommendations for interventions. An anthology book will be written as a collection of the participant's stories, the lead researcher's story, and the lead researcher's reflections of the stories. The purpose of this anthology is to disseminate the research findings to people interested in Black university student mental health, including current students and academic staff, families of students, mental health practitioners, community organisations, policy-makers, advocates, and charities. From the study findings, we aim to contribute to the evidence base on race, mental health and higher education; provide relevant recommendations for interventions; and encourage further study into Black student mental health.

Discussion

To our knowledge the current study is designed to be the first multi-site UK study to explore, from Black undergraduate and postgraduate university students' perspectives, the life events and experiences that effect their mental health and wellbeing throughout the university life-cycle. This qualitative approach has not been previously employed to understand what and how individual, institutional and structural factors within UK education systems impact the mental

For peer review only - http://bmjopen.bmj.com/site/about/guidelines.xhtml

health and wellbeing experiences and outcomes of Black university students. The usefulness of the data and subsequent recommendations are dependent on what is derived from the data. A multi-disciplinary coding team will ensure the inclusion of multiple perspectives in order to reduce researcher bias and provide opportunities to discuss disagreements in interpretations[60]. IPA is one of the appropriate methodological choices to offer insights into how a given person, in a given context, makes sense of a given phenomena[64-65]. Combining narrative inquiry with phenomenology and hermeneutics is a suitable approach to attempt to gain insight into Black students' subjective experiences of life in university, while at the same time encouraging Black students to tell their own stories.

The study findings will be limited to the time and context of this study as realities and perspectives will differ across contexts and time[66]. Interpretation of study findings will take into account potential limitations regarding the generalisability of the findings. The current project is exploring the lives of only 20-25 Black students studying at 10 UK universities, so may lack generalizability, representativeness and transferability of findings. However, in IPA research, "samples in IPA studies are usually small, which enables a detailed and very time-consuming case-by-case analysis" (pg. 4)[55]. IPA is not concerned with generating a generalisable theory, however, comparisons of multiple IPA studies on the mental health of Black university students and student mental health inequalities over time may provide insights into generalisable patterns and mechanisms[55-56]). Future research may include a mixed methods study of Black students across the educational pipeline, and inclusion of comparison groups from White student groups and other marginalised student groups, e.g., disability; Lesbian, Gay, Bisexual, Pansexual, Transgender, Genderqueer, Queer, Intersex, Agender, Asexual and other queer-identifying community (LGBTQIA+); first-generation students.

List of abbreviations

**BNIM**	Biographical Narrative Interpretive Method
**CRT**	Critical race theory
**ESRC**	Economic and Social Research Council
**GDPR**	General Data Protection Regulation

For peer review only - http://bmjopen.bmj.com/site/about/guidelines.xhtml

**HEI**	Higher Education Institute
**HERON**	Health Inequalities Research Network
**IPA**	Interpretative Phenomenological Approach
**KCL**	King's College London
**LGBTQIA+**	Lesbian, Gay, Bisexual, Pansexual, Transgender, Genderqueer, Queer, Intersex, Agender, Asexual and other queer-identifying community
**NIHR**	National Institute for Health Research
**OFS**	The Office of Students
**PINs**	Particular Incident Narratives
**SMaRteN**	The Student Mental Health Research Network
**SQUIN**	Single Question Inducing Narrative
**UK**	United Kingdom
**LISS-DTP**	London Interdisciplinary Social Science Doctoral Training Partnership
**CSMH**	Centre for Society and Mental Health

Consent for publication: Not applicable.

Data availability statement: No data are available.

Competing interests: The authors declare that they have no competing interests.

Public and Patient Involvement: The lead author is a Black Caribbean PhD student who designed and wrote up the study protocol and the second author is a Black African master's student who was involved in the design of this protocol.

Funding:

N.S. is supported by Economic and Social Research Council (ESRC) [grant number ES/P000703/1] via the London Interdisciplinary Social Science Doctoral Training Partnership (LISS-DTP) and the ESRC Centre for Society and Mental Health at KCL [ES/S012567/1].

S.H. is partly supported by the ESRC Centre for Society and Mental Health at KCL [ES/S012567/1] and by the the National Institute for Health Research (NIHR) Maudsley

For peer review only - http://bmjopen.bmj.com/site/about/guidelines.xhtml

Biomedical Research Centre at South London and Maudsley NHS Foundation Trust and King's College London [BRC-1215-20018]. The views expressed are those of the authors and not necessarily those of the NHS, the NIHR or the Department of Health and Social Care, Wellcome Trust, ESRC or KCL.

H.L. currently receives funding for successful grants as a PI or co- PI: Wellcome Trust Institutional Strategic Support Fund [No grant number]; King's Health Partner Multiple Long-Term Conditions Challenge Fund [No grant number]; National Axial Spondyloarthritis Society [No grant number]; UKRCI Medical Research Council [MR/S001255/1] and [MR/R023697/1]; NIHR [RP-PG-0610-10066]; Guy's and St. Thomas' Charity, London [EFT151101]; and Versus Arthritis [No grant number].

N.B. is partly supported by the ESRC funding for SMaRteN [ES/S00324X/1].

D.S., J.A., and Y.Y. received no funding for this research.

The funders had no involvement in the study design; collection, analysis and interpretation of the data; writing of the paper; or decision to submit the paper for publication.

For peer review only

Authors' contributions: N.S. developed the protocol, based on contribution from the other authors with respect to methodological and contextual knowledge (Y.Y., J.A., D.S., N.B., H.L., S.H.).

Acknowledgements: The authors would like to thank Dr Tom Wengraf for his contribution by reading and commenting on the initial draft of the manuscript.

Word count: 3,459 words.

References

1. Hubble, S. and Bolton, P. (2020). Support for students with mental health issues in higher education in England. Accessed from: https://commonslibrary.parliament.uk/

For peer review only - http://bmjopen.bmj.com/site/about/guidelines.xhtml

2. Office for Students (2019). Mental health: Are all students being properly supported? Accessed from: https://www.officeforstudents.org.uk/media/b3e6669e-5337-4caa-9553-049b3e8e7803/insight-brief-mental-health-are-all-students-being-properly-supported.pdf
3. Universities UK (2017). Higher Education in Facts and Figures. Accessed from: http://www.universitiesuk.ac.uk/facts-and-stats/data-and-analysis/Documents/higher-education-in-facts-and-figures-2017.pdf

4. Universities UK (2018). Minding our future: Starting a conversation about the support of student mental health. Accessed from: https://www.universitiesuk. ac.uk/policy-and-analysis/reports/Pages/minding-ourfuture-starting-a-conversation-support-student-mentalhealth.aspx
5. Wood, D., Crapnell, T., Lau, L., Bennett, A., Lotstein, D., Ferris, M., & Kuo, A. (2017). Emerging Adulthood as a Critical Stage in the Life Course. In N. Halfon (Eds.) et. al., Handbook of Life Course Health Development, 123–143. Springer.
6. Hochberg, Z. E., & Konner, M. (2020). Emerging Adulthood, a Pre-adult Life-History Stage. Frontiers in endocrinology, 10, 918. https://doi.org/10.3389/fendo.2019.00918
7. Centre for Mental Health (2016). 16-25 years Missed opportunities: children and young people's mental health. Accessed from: https://www.centreformentalhealth.org.uk/sites/default/files/2018-09/CentreforMentalHealth_MissedOpportunities_16-25years.pdf
8. Gobbi G, Atkin T, Zytynski T, et al. Association of Cannabis Use in Adolescence and Risk of Depression, Anxiety, and Suicidality in Young Adulthood: A Systematic Review and Meta-analysis. JAMA Psychiatry. 2019;76(4):426–434. doi:10.1001/jamapsychiatry.2018.4500
9. Kessler, R. C., Amminger, G. P., Aguilar-Gaxiola, S., Alonso, J., Lee, S., & Ustün, T. B. (2007). Age of onset of mental disorders: a review of recent literature. Current opinion in psychiatry, 20(4), 359–364. https://doi.org/10.1097/YCO.0b013e32816ebc8c
10. Higher Education Statistics Agency (2019). Students by level of study and academic year. Accessed from: https://www.hesa.ac.uk/data-and-analysis/students/chart-1
11. Temple, P., Callender, C., Grove, L. & Kersh, N. (2016) Managing the student experience in English higher education: differing responses to market pressures. London Review of Education, 14 (1). 33-46. DOI: 10.18546/LRE.14.1.05
12. Stallman, H. M. (2008). Prevalence of psychological distress in university students. Australian Family Physician, 37(8), 673– 677.

For peer review only

13. Friedlander, L., Reid, G., Shupak, N., & Cribbie, R. (2007). Social Support, Self-Esteem, and Stress as Predictors of Adjustment to University Among First-Year Undergraduates. Journal of College Student Development, 48(3) 259-274. https://doi.org/10.1353
14. Andrews, B. & Wilding J.M. (2004). The relation of depression and anxiety to life-stress and achievement in students. British Journal of Psychology, 95, 509-521.
15. Cooke, R., Bewick, B.M., Barkham, M., Bradley, M. & Audin, K. (2006). Measuring, monitoring and managing the psychological well-being of first year university students. British Journal of Guidance and Counselling, 34, 505-518.
16. Chung, W. W., & Hudziak, J. J. (2017). The Transitional Age Brain: "The Best of Times and the Worst of Times". Child and adolescent psychiatric clinics of North America, 26(2), 157–175. https://doi.org/10.1016/j.chc.2016.12.017).
17. Boden, J.M. & Foulds, J.A. (2016). Major depression and alcohol use disorder in adolescence: Does comorbidity lead to poorer outcomes of depression? Journal of Affective Disorders, 206, 287-293. https://doi.org/10.1016/j.jad.2016.09.004.
18. McGorry, P. D., Purcell, R., Goldstone, S., & Amminger, G. P. (2011). Age of onset and timing of treatment for mental and substance use disorders: implications for preventive intervention strategies and models of care. Current opinion in psychiatry, 24(4), 301–306. https://doi.org/10.1097/YCO.0b013e3283477a09
19. Hawton, K., Saunders, K. E., & O'Connor, R. C. (2012). Self-harm and suicide in adolescents. Lancet (London, England), 379(9834), 2373–2382. https://doi.org/10.1016/S0140-6736(12)60322-5).
20. Eskin, M., Voracek, M., Tran, T., Sun, J., Janghorbani, M., Giovanni Carta, M., … & Harlak, H. (2016). Suicidal Behavior and Psychological Distress in University Students: A 12-Nation Study. Archives of Suicide Research. Archives of Suicide Research, 20(3), 369-388. doi: 10.1080/13811118.2015.1054055
21. Waller, R., Ingram, N., & Ward, M.R.M. (Eds.). (2017). Higher Education and Social Inequalities: University Admissions, Experiences, and Outcomes (1st ed.). Routledge. https://doi.org/10.4324/9781315449722
22. Advance HE (2020). Student Inequalities. Accessed from: https://www.advance-he.ac.uk/guidance/governance/governance-and-edi/edi-challenges-higher-education/student-inequalities

For peer review only - http://bmjopen.bmj.com/site/about/guidelines.xhtml

23. Bhopal, K and Henderson, H (2019) 'Competing inequalities: Gender versus race in higher education institutions in the UK'. Educational Review. https://www.tandfonline.com/doi/abs/10.1080/00131911.2019.1642305
24. Bhopal, K (2019) 'Gender, ethnicity and career progression in UK higher education: a case study analysis'. Research Papers in Education. https://www.tandfonline.com/doi/full/10.1080/02671522.2019.1615118
25. Rollock, N. (2011) Unspoken rules of engagement: navigating racial microaggressions in the academic terrain. International Journal of Qualitative Studies in Education, 25 (5), 517-532
26. Bhopal, K (2017) 'Addressing racial inequalities in higher education: equity, inclusion and social justice'. Ethnic and Racial Studies, 40 (13), 2293-2299. DOI: 10.1080/01419870.2017.1344267
27. Alexander, C. (Ed.) (2015). Aiming Higher: Race, Inequality and Diversity in the Academy. Runnymede Trust. http://www.runnymedetrust.org/uploads/Aiming%20Higher.pdf
28. Universities UK (2019). Black, Asian and Minority Ethnic student attainment at UK universities: #closingthegap. Acessed from: https://www.universitiesuk.ac.uk/policy-and-analysis/reports/Documents/2019/bame-student-attainment-uk-universities-closing-the-gap.pdf
29. Equality Challenge Unit. 2017. Equality in Higher Education: Statistical Report. https://www.ecu.ac.uk/publications/equality-in-higher-education-statistical-report-2017/.).
30. Arday J. (2018). Understanding Mental Health: What Are the Issues for Black and Ethnic Minority Students at University? Social Sciences; 7(10):196. https://doi.org/10.3390/socsci7100196
31. Arday J. (2020) Fighting the tide: Understanding the difficulties facing Black, Asian and Minority Ethnic (BAME) Doctoral Students' pursuing a career in Academia. Educational Philosophy and Theory, DOI: 10.1080/00131857.2020.1777640
32. Arday J. (2018). Dismantling power and privilege through reflexivity: negotiating normative Whiteness, the Eurocentric curriculum and racial micro-aggressions within the Academy, Whiteness and Education, 3:2, 141-161. DOI: 10.1080/23793406.2019.1574211
33. Rollock, N. and Gillborn, D. (2011) Critical Race Theory (CRT), British Educational Research Association online resource. Available online at https://www.bera.ac.uk/publication/critical-race-theory-crt.
34. Crenshaw, K., Gotanda, N., Peller,G., & Thomas, K. (1996). Critical Race Theory: The Key Writings that Formed the Movement. The New Press. ISBN 9781565842717

For peer review only - http://bmjopen.bmj.com/site/about/guidelines.xhtml

35. Charles, H. (2008). Toward a critical race theory of education, Contemporary Justice Review. 11:1, 63-65, DOI: 10.1080/10282580701850413
36. Warmington, P. (2020). Critical race theory in England: impact and opposition, Identities, 27:1, 20-37, Doi.: 10.1080/1070289X.2019.1587907
37. Bhopal, K. & Pitkin, C. (2020) 'Same old story, just a different policy': race and policy making in higher education in the UK, Race Ethnicity and Education, 23:4, 530-547, DOI: 10.1080/13613324.2020.1718082
38. Doharty, N., Madriaga, M., & Joseph-Salisbury, R. (2020): The university went to 'decolonise' and all they brought back was lousy diversity double-speak! Critical race counter-stories from faculty of colour in 'decolonial' times, Educational Philosophy and Theory, DOI: 10.1080/00131857.2020.176960
39. Ledesma, M. C., & Calderón, D. (2015). Critical Race Theory in Education: A Review of Past Literature and a Look to the Future. Qualitative Inquiry, 21(3), 206–222. https://doi.org/10.1177/1077800414557825
40. Chakrabarty, N., Roberts, L. & Preston, J. (2012). Critical Race Theory in England, Race Ethnicity and Education, 15:1, 1-3, Doi: 10.1080/13613324.2012.638860
41. Volpe, V. V., Dawson, D. N., Rahal, D., Wiley, K. C., & Vesslee, S. (2019). Bringing psychological science to bear on racial health disparities: The promise of centering Black health through a critical race framework. Translational Issues in Psychological Science, 5(4), 302–314. https://doi.org/10.1037/tps0000205
42. Salter, P., & Adams, G. (2013). Toward a Critical Race Psychology. Social and Personality Psychology Compass, 7(11), 781–793. https://doi.org/10.1111/spc3.12068
43. Wengraf, T. (2001) Qualitative Research Interviewing: Biographic Narrative and Semi-Structured Methods. Sage Publications, London, 7. https://doi.org/10.4135/9781849209717
44. Wengraf, T. (2013). BNIM Short Guide bound with the BNIM Detailed Manual. Interviewing for life-histories, lived periods and situations, and ongoing personal experiencing using the Biographic-Narrative Interpretive Method (BNIM) Version 2013c. For a free updated version, write to tom@tomwengraf.com
45. Smith J. A. (2011). Evaluating the contribution of interpretative phenomenological analysis. Health Psychology Review, 5, 9-27.
46. Smith, J. A. (1999). Identity development during the transition to motherhood: An interpretative phenomenological analysis. Journal of Reproductive and Infant Psychology, 17, 281-300.

For peer review only - http://bmjopen.bmj.com/site/about/guidelines.xhtml

47. Smith, J. A. (2004). Reflecting on the development of interpretative phenomenological analysis and its contribution to qualitative research in psychology. Qualitative Research in Psychology, 1, 39-54.
48. Smith, J. A. (2008). Qualitative Psychology: A Practical Guide to Research Methods. London: Sage.
49. Smith JA, Flowers P, Larkin M (2009). Interpretative phenomenological Analysis: Theory, Method, Research. London: Sage
50. Smith, J. A., Harré, R., & Van Langenhove, L. (1995). Idiography. In J. A. Smith, R. Harré, & L. Van Langenhove, Rethinking Psychology, 56-69. London: Sage.
51. Smith, J. A., & Osborn, M. (2008). Interpretative Phenomenological Analysis. In J. Smith, Qualitative Psychology: A Practical Guide to Research Methods, 53-80. London: Sage.
52. Flowers, P., Smith, J., Sheeran, P. i Beail, N. (1997). Health and romance: Understanding unprotected sex in relationships between gay men. British Journal of Health Psychology, 2, 73-86.
53. Hesketh, RF and Robinson, G (2019) Grafting: "the boyz" just doing business? Deviant entrepreneurship in street gangs. Safer Communities. https://dx.doi.org/10.1108/SC-05-2019-0016
54. Malmqvist, J., Hellberg, K., Möllås, G., Rose, R., & Shevlin, M. (2019). Conducting the Pilot Study: A Neglected Part of the Research Process? Methodological Findings Supporting the Importance of Piloting in Qualitative Research Studies. International Journal of Qualitative Methods. https://doi.org/10.1177/1609406919878341
55. Pietkiewicz, I. & Smith, J.A. (2012) Praktyczny przewodnik interpretacyjnej analizy fenomenologicznej w badaniach jakościowych w psychologii. Czasopismo Psychologiczne, 18(2), 361-369
56. Smith, J. A., Harré, R., & Van Langenhove, L. (1995). Idiography. In J. A. Smith, R. Harré, & L. Van Langenhove, Rethinking Psychology, 56-69. London: Sage.
57. Shaw, R. (2010). Embedding Reflexivity Within Experiential Qualitative Psychology, Qualitative Research in Psychology, 7: 3, 233 — 243. Doi: 10.1080/14780880802699092
58. Vicary, S., Young, A., & Hicks, S. (2017). A reflective journal as learning process and contribution to quality and validity in interpretative phenomenological analysis. Qualitative Social Work, 16(4), 550–565. https://doi.org/10.1177/1473325016635244
59. Birks, M., Chapman, Y., & Francis, K. (2008). Memoing in qualitative research: Probing data and processes. Journal of Research in Nursing, 13(1), 68–75. https://doi.org/10.1177/1744987107081254

For peer review only

For peer review only - http://bmjopen.bmj.com/site/about/guidelines.xhtml

60. Berends, L. & Johnston, J. (2005). Using multiple coders to enhance qualitative analysis: The case of interviews with consumers of drug treatment. Addiction Research & Theory. 13 (4), 373-381, DOI: 10.1080/16066350500102237
61. O'Connor, C., & Joffe, H. (2020). Intercoder Reliability in Qualitative Research: Debates and Practical Guidelines. International Journal of Qualitative Methods. https://doi.org/10.1177/1609406919899220
62. Adamy, E., Zocche, D., Vendruscolo, C., Guedes dos Santos, J., & Almeida, M. (2018). Validation in grounded theory: conversation circles as a methodological strategy. Revista Brasileira de Enfermagem. 71. 3121-3126. Doi: 10.1590/0034-7167-2017-0488.
63. King's College London (2019). King's College London Records and Data Retention Schedule. Accessed from: https://www.kcl.ac.uk/aboutkings/orgstructure/ps/audit/records/retention/rdrs-v7.pdf
64. Alase, A. (2017). The Interpretative Phenomenological Analysis (IPA): A Guide to a Good Qualitative Research Approach. International Journal of Education and Literacy Studies, 5(2), 9-19. doi:http://dx.doi.org/10.7575/aiac.ijels.v.5n.2p.9
65. Creswell, J. W., & Poth, C. N. (2018). Qualitative inquiry & research design: Choosing among five approaches.
66. Austin, Z., & Sutton, J. (2014). Qualitative research: getting started. The Canadian journal of hospital pharmacy, 67(6), 436–440. https://doi.org/10.4212/cjhp.v67i6.1406

For peer review only

BMJ Open

Protocol for Black Student Wellbeing Study: A multi-site qualitative study on the mental health and wellbeing experiences of Black UK university students

Journal:	*BMJ Open*
Manuscript ID	bmjopen-2021-051818.R1
Article Type:	Protocol
Date Submitted by the Author:	21-Nov-2021
Complete List of Authors:	Stoll, Nkasi; Institute of Psychiatry Psychology and Neuroscience Division of Academic Psychiatry, Psychological medicine; King's College London, Centre For Society and Mental Health<br>Yalipende, Yannick; University College London, Division of Psychology and Language Sciences<br>Arday, Jason; University of Durham Department of Sociology and Social Policy, Higher Education and Social Inequality<br>Smithies , Dominic; Student Minds<br>Byrom, Nicola; King's College London, Department of Psychology<br>Lempp, Heidi; King’s College London, Inflammation Biology<br>HATCH, STEPHANI; Institute of Psychiatry Psychology and Neuroscience Division of Academic Psychiatry, Psychological medicine; King's College London, Centre For Society and Mental Health
**Primary Subject Heading**:	Qualitative research
Secondary Subject Heading:	Mental health, Public health, Research methods, Sociology, Epidemiology
Keywords:	MENTAL HEALTH, QUALITATIVE RESEARCH, PSYCHIATRY, Health policy < HEALTH SERVICES ADMINISTRATION & MANAGEMENT, EDUCATION & TRAINING (see Medical Education & Training)

SCHOLARONE™
Manuscripts

Protocol for Black Student Wellbeing Study: A multi-site qualitative study on the mental health and wellbeing experiences of Black UK university students

Ms Nkasi Stoll[1,2] Mr Yannick Yalipende[3], Dr Jason Arday[4], Mr Dominic Smithies[5], Dr Nicola Byrom[6], Professor Heidi Lempp[7]*, Professor Stephani L Hatch[1,2]*

[1]King's College London, Institute of Psychiatry Psychology and Neuroscience, Department of Psychological Medicine, London, UK.

[2]King's College London, ESRC Centre for Society and Mental Health, London, UK.

[3]University College London, Division of Psychology and Language Sciences, London, UK.

[4]Durham University, Department of Sociology, Higher Education and Social Inequality, UK.

[5]Student Minds, the UK's student mental health charity, 17 Springfield Mount, Woodhouse, Leeds LS2 9NG.

[6]King's College London, Institute of Psychiatry Psychology and Neuroscience, Department of Psychology, London, UK.

[7]King's College London; Faculty of Life Sciences and Medicine, Department of Inflammation Biology, Centre for Rheumatic Diseases, London UK.

Corresponding Author:

Ms Nkasi Stoll, BSc, MSc

King's College London, Institute of Psychiatry Psychology and Neuroscience

Department of Psychological Medicine

16 De Crespigny Park

London, SE5 8AF, UK

Email: Nkasi.1.stoll@kcl.ac.uk

Twitter: @NkasiStoll

For peer review only

Abstract

Introduction: There is increasing concern about the mental health and mental wellbeing of university students in the United Kingdom (UK). Black university students who report a mental health condition are less likely to complete their course, achieve a first-class or upper second-class degree, and progress to further education. This study will document Black university students' accounts of their mental health experiences and perceptions of key turning points of biographical changes to their mental health as they move through the university life cycle.

Methods and Analysis: This is a qualitative study. Data will be collected through a Biographical Narrative Interpretive Method. Interviews will enable the researcher to study systematically how participants make sense of themselves and account for the complexities of their life experiences, from their own perspectives and language. An interpretative Phenomenological Approach will be used to offer insights into what Black students studying at UK universities report affects their mental health and wellbeing. Data collection for this study commenced in October 2020.Data collection and analyses will be completed by January 2022.

Ethics and Dissemination: Full ethical approval for the current study was obtained from King's College London Psychiatry, Nursing and Midwifery Research Ethics Subcommittee (Rec Ref: 20489, Project Ref: HR-19/20-20489, 2nd October 2020). From the study findings, we aim to contribute to the evidence base, make recommendations for interventions, and encourage further study into Black student mental health.

For peer review only

Strengths and Limitations of this study

- The current study is designed to be the first UK national multi-site study to explore, from Black undergraduate and postgraduate university students' perspectives, the life events and experiences that effect their mental health and wellbeing throughout the university life-cycle.
- A multi-disciplinary coding team (including Black university students) will ensure the inclusion of multiple perspectives to reduce researcher bias and provide opportunities to discuss disagreements in interpretations.

For peer review only - http://bmjopen.bmj.com/site/about/guidelines.xhtml

- This study will contribute to the evidence base on race, mental health and higher education; provide relevant recommendations for interventions; and encourage further study into Black student mental health.
- The current project is exploring the lives of 20-25 Black students studying at 10 UK universities, so may lack generalizability, representativeness and transferability of findings.

Introduction

The mental health and mental wellbeing of university students in the United Kingdom (UK) is of increasing concern to policymakers, researchers, charities, university staff and students[1-4]. Young adulthood is a critical age for emotional, psychosocial and cognitive development[5-6] and first onset of mental health problems, suicidality, and substance misuse problems[7-9]. Young adulthood is also the time that many enter and navigate university in the UK. There are approximately 2.4 million university students in the UK, the majority being young adults[10], which means Higher Education Institutes (HEI) are in the best position to provide prevention and intervention to many young adults during an important transitional period.

Throughout the university life-cycle[11], students are exposed to a range of experiences that make higher education a high-risk period for maladaptive coping and possible onset of mental health problems, including (but not limited to): individuation, separation from family, , increased autonomy and responsibility, academic-related stress, financial concerns, sleep disruption, balancing conflicting demands of studying with personal and family life, and exposure to risky behaviours, including recreational drug use and alcohol binging[12-16]. Unsupported mental health problems are associated with progression to other co-morbid disorders, substance use disorders, self-harm and suicide ideation and attempts[17-20].

A growing body of evidence explores inequalities and inequity in student admissions, experiences and outcomes in UK HEIs by race, gender, class and (dis)ability[21-27]. Black students with a mental health problem studying at UK HEI are less likely to complete their course, achieve a first- or upper second- class degree, and progress to further education[28-29]. The Office for Students[2] reported that "black students with mental health conditions are being

For peer review only - http://bmjopen.bmj.com/site/about/guidelines.xhtml

failed throughout the student cycle" (pg. 6), but that there is limited data or research available to explain how and why. Available research indicates the following factors and dimensions may explain the inequality and inequity observed in higher education, at the expense of Black students: racism and structural racism, discrimination, mental health stigma, sexism, cultural incompetence and insensitivity of healthcare professionals[30-32]. It is essential to know more about the experiences of, risk factors for, and challenges that affect Black university students' mental health in UK higher education in order to (re-)design, develop and deliver appropriate mental health and wellbeing services.

Critical Race Theory

Critical race theory (CRT) offers an interpretive theoretical approach to explore and challenge racial inequality in society. It is based on the understanding that racism is embedded as normal practice within society and institutions, rooted in slavery and colonialism; and race is a socially constructed concept that is used by White people to further their socio-economic and political interests and power at the expense of racialised minorities. This racial bias causes discrimination within law, employment, housing, healthcare, politics, and education, disadvantaging racialised minorities[33-34]. CRT in the educational context explores and challenges the White supremacist patriarchal structures and assumptions that have historically shaped education[33,35]. CRT theorists explore racial inequality in: admissions, curriculum and pedagogy, teaching and learning, institutional culture, campus racial climate, and policy and finance within UK further and higher education[36-40]. CRT is also used as a conceptual lens through which to interrogate the generational impact slavery and colonialism has on an individuals and families, as well as psychological research and practices, at the expense of racialised minorities[41-42]. Therefore, CRT will guide the direction of this current study by providing a framework to support the study team to interrogate the methodology, analyses, and interpretation throughout the research process[32].

Aims

The aim of this study is to document and explore Black university students' (i) accounts of their mental health experiences as they transition throughout their university lives; and (ii) perceptions of key turning points of biographical change to their mental health in their lives as they move through the university student life cycle.

For peer review only

Objectives

This study uses Biographic Narrative Interpretive Method (BNIM)[43-44] interviews and Interpretative Phenomenological Approach (IPA)[45-52] to get an in-depth understanding of the educational and mental health of experiences Black UK university students.

Research questions

1. From the students' perspective, what life events and experiences do Black university students perceive affect their mental health and wellbeing?
2. How does institutional and structural racism within UK education systems affect the mental health and wellbeing experiences and outcomes of Black university students?

Methods and Analysis

Study design

This is a qualitative study. Data is being collected through BNIM interviews[43-44]. This interview technique enables the researcher to study how participants make sense of themselves and account for the complexities of their life experiences, from their own perspectives and choice of language[43-44]. For the current study, IPA[45-52] offers insights into what Black students studying at UK universities report affects their mental health and wellbeing. Data collection for this study commenced in October 2020. Data collection and analyses are planned to be completed by January 2022.

Recruitment

A webpage was set up specifically for the study on the Health Inequalities Research Network (HERON) website: www.heronnetwork.com which is General Data Protection Regulation (GDPR) complaint. The webpage has the lead researcher (N.S.)'s contact information, the study's rationale, expression of interest form, information sheet, consent form, list of support services, and updates about the timeline of the project.. The study is advertised on social media accounts (Twitter and Instagram) set up specifically for the study. Social media posts with a short summary of the study were designed and sent out on this social media page to recruit participants. The social media posts include the study's website and QR code which means that

For peer review only

potential participants can access the webpage with one click. Recruitment emails were sent to student unions, student groups and student services..

Completed expression of interest forms are automatically sent to a KCL study mailbox which was set up for the study. N.S. monitors the incoming forms twice a day and screens the participants based on the inclusion and exclusion criteria. Potential participants are contacted by N.S. via email to complete an attached consent form which they are asked to email back to the researcher. Participants have a minimum of 48 hours to decide whether they would like to participate in the study. If the participant has not responded after two days they are sent a reminder email by N.S. and subsequent two reminder emails until they are considered non-responsive.

Sample size

Purposive sampling[51] is being used to recruit Black university students who meet the inclusion criteria. 15-20 students are being recruited from nine Russell and non-Russell group universities across the UK. Data from the Higher Education Statistics Agency (HESA)[10] for UK higher education student enrolments by higher education provider and ethnicity for the academic years 2014/15 to 2018/19 was used to decide which universities to recruit from. In Microsoft Excel the data was sorted into higher education providers with the highest percentage of Black students enrolled, to the lowest. After this, inter-quartile range was calculated to divide the higher education providers into quartiles of median, lower (i.e., lowest percentage of Black students) and upper range (i.e., highest percentage of Black students). Three higher education providers that fall into each range were chosen.

Sample

Two universities are in Ireland, one university is in Scotland, and seven universities are in England. Of the universities based in England, three are in London, one is in the East Midlands, one university in Southeast England, one in the West Midlands, and the final in East of England. Five universities are Russell Group universities and five are non-Russell Group universities. Russell group universities are defined as “a group of unis with a shared focus on research and a reputation for academic achievement”[53]. Whilst data from the National Student

For peer review only - http://bmjopen.bmj.com/site/about/guidelines.xhtml

Surveys suggest there are no statistically significant differences in student satisfaction between Russell and non-Russell group universities[54]. There is some evidence that ethnic minority applicants to Russell Group universities may be less likely to receive offers to study, compared to similarly qualified white applicants[55]. Six universities are based in the city where the campuses and facilities are spread out across the city, and four universities are campus universities, situated in rural settings.

Inclusion and Exclusion criteria

The inclusion criteria for students are deliberately broad to include students who: (1) self-identify as Black (Caribbean, African, and mixed heritage with Black); (2) are currently studying at, within a year of graduating from, or are within a year of dropping out of a university course; (3) self-identify as having struggled with their mental health and/or wellbeing whilst studying at a UK university; (4) are aged 18 or over. As an IPA study[45-52], this will allow for a wide range of Black university students, from different backgrounds, to be eligible for study inclusion as the study processes.

The exclusion criteria are people who: (1) have never been enrolled on a course at a UK university, (2) are enrolled at a university outside of the UK, (3) do not identify as Black (Caribbean, African, and mixed heritage with Black), (4) graduated or dropped out of university over a year ago, (5) are aged 17 or younger.

Potential participants are asked to complete an expression of interest form online, with sociodemographic information to determine their eligibility for the study.

Data collection

Data collection for this study commenced in October 2020. Data collection and analyses will be completed by January 2022. All interviews are being conducted by N.S. In order to generate a narration that allows the interviewee to begin, construct and end their narrative on their own terms , students are invited to participate in a hybrid adapted version of (BNIM)[43-44] designed by N.S. in collaboration with the authors. The BNIM approach to data gathering is an open narrative interview process where theinterviewer is encouraged to participate in active

For peer review only

listening, and not interrupt with questions. The students are asked a single question called a 'Single Question Inducing Narrative' (SQUIN)[43-44] to describe their life, in terms of their mental health and wellbeing. The SQUIN reads as follows: *"As you know, I am researching mental health and wellbeing experiences of Black university students in the UK. I understand that you have had such experiences. So please can you tell me the story of your mental health experiences before university and whilst studying at university. Please include all the events and experiences that were important for you, personally, up until now. There's no rush, you can start wherever you like. We've got 2 hours in total. I'll listen first, I won't interrupt. I'll tell you if we are running out of time. I'll just take some notes and when you've finished telling me about it all we will have a 15-minute break. After the break I may ask you further questions about your story".* As the student is talking, N.S. takes notes on what Wengraf terms 'Particular Incident Narratives' (PINs)[43-44], which are significant events that have occurred in each student's life surrounding their mental health and wellbeing using the keywords that they use. Students are then invited to take a 15-minute break and during this time N.S. identifies questions called narrative/N-pointed questions[43-44], to clarify or pick up on PINs[43-44]. The second session begins with N.S. asking the student to reflect back on the identified PINs and continue with the N-pointed questions. The interview ends with the question: "Before we finish, is there anything you would like to add that we didn't talk about?". Interviews last up to 2 hours in total. The interviews elicit two transcripts of narrative for data analysis from each student.

Interviews are conducted virtually using Microsoft Teams and are recorded with the student's permission. N.S. transcribes the interviews by hand using Microsoft Teams' automated speech-to-text transcript programme, before analysis.

A BNIM pilot study was conducted to assess comprehension of the BNIM question, relevance, timing, and research burden[56]. Two Black University students were invited to participate in the pilot study. Data was presented to the fifth, sixth, and seventh authors of this paper to collaboratively agree whether they matched the study objectives. No amendments were made to the study.

For peer review only - http://bmjopen.bmj.com/site/about/guidelines.xhtml

Data analysis

Data analysis began after the first interview in October 2020. Data is being analysed using IPA[45-52] on NVivo Qualitative Data Analysis Software (version 12). An inductive analysis of each student's account is conducted before moving onto group analysis[47]. Analysis began with multiple readings of each student's transcript by N.S., who makes notes on the student's language and structure (including repetitions, exaggerations, emphasis, symbols, pauses, slang), emotional responses, and any insights into the students' experience and perspective on their world i.e., 'codes'. This is to interpret what and how the student is communicating their story. As the analysis develops, N.S. looks for patterns in the codes to create emerging themes. Themes identify recurring patterns of meaning (ideas, thoughts, feelings, topics of importance or concern) presented by the student and interpreted by N.S. throughout the text. Themes will eventually be linked and grouped together to create subordinate themes. The final set of themes will be summarised, arranged into clusters to form master themes and placed into a table. Quotations from the students will be selected to illustrate each theme. All the transcripts will be collated and re-examined by N.S. who will pay attention to thematic overlap and differences in student's accounts and will make links to existing psychological and sociological literature, concepts and theories which provide an in-depth understanding of the phenomena and strengthen the robustness of the data analysis. The authors will be careful to apply a critical race lens on the existing theoretical knowledge within this area of research to explain the phenomena[55].

In IPA "the participants are trying to make sense of their world; the researcher is trying to make sense of the participants trying to make sense of their world" (pg. 53[51]). The dialogue between the researcher and the participant is described as a double hermeneutic and IPA recognises the importance of constant self-awareness and reflexivity[57]. N.S. writes notes in a reflective diary throughout study conception to dissemination to draw their observations and reflections about the interview experience, data analysis and interpretations; or any other thoughts and comments of potential significance[57-59].

A second coder, a Black university student (Y.Y.), will code 20% of the interviews using IPA for cross-validation of the themes[60-61]. The authors of this paper will form a multi-disciplinary coding team and participate in conversation circles over Microsoft Teams to validate the data, participate in theme development, and promote reflexivity and dialogue[60,62]. The authors all

For peer review only - http://bmjopen.bmj.com/site/about/guidelines.xhtml

have extensive knowledge and experience on student mental health and mental health inequality in the UK. The circle mediator (N.S.) will participate in the conversation to present and clarify ideas or issues and encourage the dialogue. The conversation circle will be audio recorded with verbal consent of the attendees. During the conversation the objectives, methodological process, and research results will be presented. The authors will be encouraged, in a dialogic process, to give their opinion about the codes and emergent themes. A second conversation circle will be arranged for the subordinate themes.

Data protection

All confidential data is kept for 10 years according to KCL Records and Data Retention Schedule (2019)[63]. Data is stored on password-protected computers in password protected, encrypted folders on KCL SharePoint which are only identifiable by the code number and only accessible to members of the research team. During transcription, audio-recordings are anonymised, with all identifiable information removed prior to using NVivo Software. All audio-recordings are destroyed immediately after transcription. This is important to ensure that their identities are protected, and they are not at risk of harm due to damage of their reputation or social standing for example, should they express controversial or negative opinions or experiences. This is particularly relevant considering the sensitive topic of study and the fact that participants are invited to consider current as well as historical experiences at university.

Public and Patient Involvement

Public and Patient Involvement: The lead author is a Black Caribbean PhD student who designed and wrote up the study protocol and the second author is a Black African master's student who was involved in the design of this protocol.

For peer review only

Ethics and Dissemination

Full ethical approval has been granted by KCL Psychiatry, Nursing and Midwifery Research Ethics Subcommittee (Rec Ref: 20489, Project Ref: HR-19/20-20489, 2nd October 2020). Written informed consent to participate and audio-recorded interviews is obtained from all participants. Participants consent to the inclusion of material pertaining to themselves, that they acknowledge they cannot be identified via the manuscript; and that the participant has been fully anonymised by the author. Data management and storage is subject to the UK Data Protection Act 1998 and GDPR. The authors assert that all procedures contributing to this work comply with the ethical standards of the relevant national and institutional committees on human experimentation and with the Helsinki Declaration of 1975, as revised in 2008.

Safety Considerations

Potential risks of participating come as a result of the participant disclosing a historical or current distressing experience. During the consent process and at the beginning of the interview, the interviewer (N.S.) informs each participant that confidentiality needs to be broken if they disclose personal details which raises concerns to N.S. about their safety, the safety of others, safety of someone under the age of 18 or about a previously non-disclosed serious crime they or someone else has committed or is about to commit.

When a participant discloses a serious immediate risk, the interview is stopped, a conversation is had with the participant (where appropriate) to ensure they understand why and how information is shared for their safety, and the emergency services is contacted immediately. The co-senior author (S.H.) is informed immediately after (in person, or by phone if this is not possible). Incidents are reported to the Chair of the Ethics Committee.

When a participant becomes distressed during the interview N.S. asks the participant if they would like a break. When the participant would like to terminate the interview or N.S. decides the participant is no longer able to continue she offers to re-schedule the interview at the next convenient time.

Time is explicitly allocated to a 'debrief' period at the end of the interview for all participants to reflect upon the session, ask any questions or raise anything that may be of concern. Participants are asked: "Is there anything you would like to share regarding your experience

For peer review only - http://bmjopen.bmj.com/site/about/guidelines.xhtml

talking to me today?". A list of support services available across the UK are listed in a document created for this research project, which is available on the research website in case participants need professional support. Every participant is sent an email directing them to this list of support services after the interview.

Output and Dissemination

Findings will be disseminated via an internal report (doctoral thesis), peer-reviewed journal articles (e.g., commentary piece(s), theoretical article(s), write up of the main findings) and conference presentations (e.g. Advance HE, The Student Mental Health Research Network [SMaRteN], AMOSHEE, The Office of Students [OFS]). The study team will seek funding from the London Interdisciplinary Social Science Doctoral Training Partnership (LISS-DTP), KCL, and the ESRC Centre for Society and Mental Health (CSMH) to design and deliver internal and external public engagement events to discuss the study findings and potential recommendations for interventions. An anthology book will be written as a collection of the participant's stories, the lead researcher's story, and the lead researcher's reflections of the stories. The purpose of this anthology is to disseminate the research findings to people interested in Black university student mental health, including current students and academic staff, families of students, mental health practitioners, community organisations, policy-makers, advocates, and charities. From the study findings, we aim to contribute to the evidence base on race, mental health and higher education; provide relevant recommendations for interventions; and encourage further study into Black student mental health.

Discussion

To our knowledge the current study is designed to be the first multi-site UK study to explore, from Black undergraduate and postgraduate university students' perspectives, the life events and experiences that effect their mental health and wellbeing throughout the university life cycle. This qualitative approach has not been previously employed to understand what and how individual, institutional and structural factors within UK education systems impact the mental health and wellbeing experiences of Black university students. The usefulness of the data and subsequent recommendations are dependent on what is derived from the data. A multi-disciplinary coding team will ensure the inclusion of multiple perspectives in order to reduce researcher bias and provide opportunities to discuss disagreements in interpretations[60]. IPA is

For peer review only

one of the appropriate methodological choices to offer insights into how a person, in a particular context, makes sense of a given phenomena[64-65]. Combining narrative inquiry with phenomenology and hermeneutics is a suitable approach to attempt to gain insight into Black students' subjective experiences of life in university, while at the same time encouraging Black students to tell their own stories and have their voices heard.

The study findings will be limited to the time and context of this study as realities and perspectives will differ across contexts and time[66]. Interpretation of study findings will take into account potential limitations regarding the generalisability of the findings. The current project is exploring the lives of 20-25 Black students studying at 10 UK universities, so the findings are not generalizable, representative and transferable to all Black university students studying at UK HEIs . However, in IPA research, "samples in IPA studies are usually small, which enables a detailed and very time-consuming case-by-case analysis" (pg. 4)[57]. IPA is not concerned with generating a generalisable theory, however, comparisons of multiple IPA studies on the mental health of Black university students and student mental health inequalities over time may provide insights into generalisable patterns and mechanisms[51, 57]). Future research may include a mixed methods study of Black students across the UK educational pipeline, as well as further exploration of the mental health experiences of Black students who face multiple marginalisation e.g., disabled; first-generation; carers; and Lesbian, Gay, Bisexual, Transgender, and other queer-identifying community (LGBT+) Black students..

For peer review only

List of abbreviations

**BNIM**	Biographical Narrative Interpretive Method
**CRT**	Critical race theory
**ESRC**	Economic and Social Research Council
**GDPR**	General Data Protection Regulation
**HEI**	Higher Education Institute
**HERON**	Health Inequalities Research Network
**IPA**	Interpretative Phenomenological Approach
**KCL**	King's College London

For peer review only - http://bmjopen.bmj.com/site/about/guidelines.xhtml

**LGBTQIA+**	Lesbian, Gay, Bisexual, Pansexual, Transgender, Genderqueer, Queer, Intersex, Agender, Asexual and other queer-identifying community
**NIHR**	National Institute for Health Research
**OFS**	The Office of Students
**PINs**	Particular Incident Narratives
**SMaRteN**	The Student Mental Health Research Network
**SQUIN**	Single Question Inducing Narrative
**UK**	United Kingdom
**LISS-DTP**	London Interdisciplinary Social Science Doctoral Training Partnership
**CSMH**	Centre for Society and Mental Health

Consent for publication: Not applicable.

Data availability statement: No data are available.

Competing interests: The authors declare that they have no competing interests.

Public and Patient Involvement: The lead author is a Black Caribbean PhD student who designed and wrote up the study protocol and the second author is a Black African master's student who was involved in the design of this protocol.

Funding:
N.S. is supported by Economic and Social Research Council (ESRC) [grant number ES/P000703/1] via the London Interdisciplinary Social Science Doctoral Training Partnership (LISS-DTP) and the ESRC Centre for Society and Mental Health at KCL [ES/S012567/1].

S.H. is partly supported by the ESRC Centre for Society and Mental Health at KCL [ES/S012567/1] and by the the National Institute for Health Research (NIHR) Maudsley Biomedical Research Centre at South London and Maudsley NHS Foundation Trust and King's College London [BRC-1215-20018]. SH also currently received funding from the Wellcome Trust [203380/Z/16/Z] and the Economic and Social Research Council (ESRC) [ES/V009931/1]. The

For peer review only

views expressed are those of the authors and not necessarily those of the NHS, the NIHR or the Department of Health and Social Care, Wellcome Trust, ESRC or KCL.

H.L. currently receives funding for successful grants as a PI or co- PI: Wellcome Trust Institutional Strategic Support Fund [No grant number]; King's Health Partner Multiple Long-Term Conditions Challenge Fund [No grant number]; National Axial Spondyloarthritis Society [No grant number]; UKRCI Medical Research Council [MR/S001255/1] and [MR/R023697/1]; NIHR [RP-PG-0610-10066]; Guy's and St. Thomas' Charity, London [EFT151101]; and Versus Arthritis [No grant number].

N.B. is partly supported by the ESRC funding for SMaRteN [ES/S00324X/1].

D.S., J.A., and Y.Y. received no funding for this research.

The funders had no involvement in the study design; collection, analysis and interpretation of the data; writing of the paper; or decision to submit the paper for publication.

Authors' contributions: N.S. conceived, designed, and wrote the protocol. S.H., H.L. and N.B. were involved in the design, supervision, and contributed to the final manuscript. Y.Y., D.S. and J.A. contributed to the final manuscript.

Acknowledgements: The authors would like to thank Dr Tom Wengraf for his contribution by reading and commenting on the initial draft of the manuscript.

Word count: 3,459 words.

References

1. Hubble, S. and Bolton, P. (2020). Support for students with mental health issues in higher education in England. Accessed from: https://commonslibrary.parliament.uk/
2. Office for Students (2019). Mental health: Are all students being properly supported? Accessed from: https://www.officeforstudents.org.uk/media/b3e6669e-5337-4caa-9553-049b3e8e7803/insight-brief-mental-health-are-all-students-being-properly-supported.pdf

For peer review only - http://bmjopen.bmj.com/site/about/guidelines.xhtml

3. Universities UK (2017). Higher Education in Facts and Figures. Accessed from: http://www.universitiesuk.ac.uk/facts-and-stats/data-and-analysis/Documents/higher-education-in-facts-and-figures-2017.pdf
4. Universities UK (2018). Minding our future: Starting a conversation about the support of student mental health. Accessed from: https://www.universitiesuk. ac.uk/policy-and-analysis/reports/Pages/minding-ourfuture-starting-a-conversation-support-student-mentalhealth.aspx
5. Wood, D., Crapnell, T., Lau, L., Bennett, A., Lotstein, D., Ferris, M., & Kuo, A. (2017). Emerging Adulthood as a Critical Stage in the Life Course. In N. Halfon (Eds.) et. al., Handbook of Life Course Health Development, 123–143. Springer.
6. Hochberg, Z. E., & Konner, M. (2020). Emerging Adulthood, a Pre-adult Life-History Stage. Frontiers in endocrinology, 10, 918. https://doi.org/10.3389/fendo.2019.00918
7. Centre for Mental Health (2016). 16-25 years Missed opportunities: children and young people's mental health. Accessed from: https://www.centreformentalhealth.org.uk/sites/default/files/2018-09/CentreforMentalHealth_MissedOpportunities_16-25years.pdf
8. Gobbi G, Atkin T, Zytynski T, et al. Association of Cannabis Use in Adolescence and Risk of Depression, Anxiety, and Suicidality in Young Adulthood: A Systematic Review and Meta-analysis. JAMA Psychiatry. 2019;76(4):426–434. doi:10.1001/jamapsychiatry.2018.4500
9. Kessler, R. C., Amminger, G. P., Aguilar-Gaxiola, S., Alonso, J., Lee, S., & Ustün, T. B. (2007). Age of onset of mental disorders: a review of recent literature. Current opinion in psychiatry, 20(4), 359–364. https://doi.org/10.1097/YCO.0b013e32816ebc8c
10. Higher Education Statistics Agency (2019). Students by level of study and academic year. Accessed from: https://www.hesa.ac.uk/data-and-analysis/students/chart-1
11. Temple, P., Callender, C., Grove, L. & Kersh, N. (2016) Managing the student experience in English higher education: differing responses to market pressures. London Review of Education, 14 (1). 33-46. DOI: 10.18546/LRE.14.1.05
12. Stallman, H. M. (2008). Prevalence of psychological distress in university students. Australian Family Physician, 37(8), 673– 677.
13. Friedlander, L., Reid, G., Shupak, N., & Cribbie, R. (2007). Social Support, Self-Esteem, and Stress as Predictors of Adjustment to University Among First-Year Undergraduates. Journal of College Student Development, 48(3) 259-274. https://doi.org/10.1353

For peer review only - http://bmjopen.bmj.com/site/about/guidelines.xhtml

14. Andrews, B. & Wilding J.M. (2004). The relation of depression and anxiety to life-stress and achievement in students. British Journal of Psychology, 95, 509-521.
15. Cooke, R., Bewick, B.M., Barkham, M., Bradley, M. & Audin, K. (2006). Measuring, monitoring and managing the psychological well-being of first year university students. British Journal of Guidance and Counselling, 34, 505-518.
16. Chung, W. W., & Hudziak, J. J. (2017). The Transitional Age Brain: "The Best of Times and the Worst of Times". Child and adolescent psychiatric clinics of North America, 26(2), 157–175. https://doi.org/10.1016/j.chc.2016.12.017).
17. Boden, J.M. & Foulds, J.A. (2016). Major depression and alcohol use disorder in adolescence: Does comorbidity lead to poorer outcomes of depression? Journal of Affective Disorders, 206, 287-293. https://doi.org/10.1016/j.jad.2016.09.004.
18. McGorry, P. D., Purcell, R., Goldstone, S., & Amminger, G. P. (2011). Age of onset and timing of treatment for mental and substance use disorders: implications for preventive intervention strategies and models of care. Current opinion in psychiatry, 24(4), 301–306. https://doi.org/10.1097/YCO.0b013e3283477a09
19. Hawton, K., Saunders, K. E., & O'Connor, R. C. (2012). Self-harm and suicide in adolescents. Lancet (London, England), 379(9834), 2373–2382. https://doi.org/10.1016/S0140-6736(12)60322-5).
20. Eskin, M., Voracek, M., Tran, T., Sun, J., Janghorbani, M., Giovanni Carta, M., … & Harlak, H. (2016). Suicidal Behavior and Psychological Distress in University Students: A 12-Nation Study. Archives of Suicide Research. Archives of Suicide Research, 20(3), 369-388. doi: 10.1080/13811118.2015.1054055
21. Waller, R., Ingram, N., & Ward, M.R.M. (Eds.). (2017). Higher Education and Social Inequalities: University Admissions, Experiences, and Outcomes (1st ed.). Routledge. https://doi.org/10.4324/9781315449722
22. Advance HE (2020). Student Inequalities. Accessed from: https://www.advance-he.ac.uk/guidance/governance/governance-and-edi/edi-challenges-higher-education/student-inequalities
23. Bhopal, K and Henderson, H (2019) 'Competing inequalities: Gender versus race in higher education institutions in the UK'. Educational Review. https://www.tandfonline.com/doi/abs/10.1080/00131911.2019.1642305

For peer review only

24. Bhopal, K (2019) 'Gender, ethnicity and career progression in UK higher education: a case study analysis'. Research Papers in Education. https://www.tandfonline.com/doi/full/10.1080/02671522.2019.1615118
25. Rollock, N. (2011) Unspoken rules of engagement: navigating racial microaggressions in the academic terrain. International Journal of Qualitative Studies in Education, 25 (5), 517-532
26. Bhopal, K (2017) 'Addressing racial inequalities in higher education: equity, inclusion and social justice'. Ethnic and Racial Studies, 40 (13), 2293-2299. DOI: 10.1080/01419870.2017.1344267
27. Alexander, C. (Ed.) (2015). Aiming Higher: Race, Inequality and Diversity in the Academy. Runnymede Trust. http://www.runnymedetrust.org/uploads/Aiming%20Higher.pdf
28. Universities UK (2019). Black, Asian and Minority Ethnic student attainment at UK universities: #closingthegap. Acessed from: https://www.universitiesuk.ac.uk/policy-and-analysis/reports/Documents/2019/bame-student-attainment-uk-universities-closing-the-gap.pdf
29. Equality Challenge Unit. 2017. Equality in Higher Education: Statistical Report. https://www.ecu.ac.uk/publications/equality-in-higher-education-statistical-report-2017/.).
30. Arday J. (2018). Understanding Mental Health: What Are the Issues for Black and Ethnic Minority Students at University? Social Sciences; 7(10):196. https://doi.org/10.3390/socsci7100196
31. Arday J. (2020) Fighting the tide: Understanding the difficulties facing Black, Asian and Minority Ethnic (BAME) Doctoral Students' pursuing a career in Academia. Educational Philosophy and Theory, DOI: 10.1080/00131857.2020.1777640
32. Arday J. (2018). Dismantling power and privilege through reflexivity: negotiating normative Whiteness, the Eurocentric curriculum and racial micro-aggressions within the Academy, Whiteness and Education, 3:2, 141-161. DOI: 10.1080/23793406.2019.1574211
33. Rollock, N. and Gillborn, D. (2011) Critical Race Theory (CRT), British Educational Research Association online resource. Available online at https://www.bera.ac.uk/publication/critical-race-theory-crt.
34. Crenshaw, K., Gotanda, N., Peller,G., & Thomas, K. (1996). Critical Race Theory: The Key Writings that Formed the Movement. The New Press. ISBN 9781565842717
35. Charles, H. (2008). Toward a critical race theory of education, Contemporary Justice Review. 11:1, 63-65, DOI: 10.1080/10282580701850413

For peer review only - http://bmjopen.bmj.com/site/about/guidelines.xhtml

36. Warmington, P. (2020). Critical race theory in England: impact and opposition, Identities, 27:1, 20-37, Doi.: 10.1080/1070289X.2019.1587907
37. Bhopal, K. & Pitkin, C. (2020) 'Same old story, just a different policy': race and policy making in higher education in the UK, Race Ethnicity and Education, 23:4, 530-547, DOI: 10.1080/13613324.2020.1718082
38. Doharty, N., Madriaga, M., & Joseph-Salisbury, R. (2020): The university went to 'decolonise' and all they brought back was lousy diversity double-speak! Critical race counter-stories from faculty of colour in 'decolonial' times, Educational Philosophy and Theory, DOI: 10.1080/00131857.2020.176960
39. Ledesma, M. C., & Calderón, D. (2015). Critical Race Theory in Education: A Review of Past Literature and a Look to the Future. Qualitative Inquiry, 21(3), 206–222. https://doi.org/10.1177/1077800414557825
40. Chakrabarty, N., Roberts, L. & Preston, J. (2012). Critical Race Theory in England, Race Ethnicity and Education, 15:1, 1-3, Doi: 10.1080/13613324.2012.638860
41. Volpe, V. V., Dawson, D. N., Rahal, D., Wiley, K. C., & Vesslee, S. (2019). Bringing psychological science to bear on racial health disparities: The promise of centering Black health through a critical race framework. Translational Issues in Psychological Science, 5(4), 302–314. https://doi.org/10.1037/tps0000205
42. Salter, P., & Adams, G. (2013). Toward a Critical Race Psychology. Social and Personality Psychology Compass, 7(11), 781–793. https://doi.org/10.1111/spc3.12068
43. Wengraf, T. (2001) Qualitative Research Interviewing: Biographic Narrative and Semi-Structured Methods. Sage Publications, London, 7. https://doi.org/10.4135/9781849209717
44. Wengraf, T. (2013). BNIM Short Guide bound with the BNIM Detailed Manual. Interviewing for life-histories, lived periods and situations, and ongoing personal experiencing using the Biographic-Narrative Interpretive Method (BNIM) Version 2013c. For a free updated version, write to tom@tomwengraf.com
45. Smith J. A. (2011). Evaluating the contribution of interpretative phenomenological analysis. Health Psychology Review, 5, 9-27.
46. Smith, J. A. (1999). Identity development during the transition to motherhood: An interpretative phenomenological analysis. Journal of Reproductive and Infant Psychology, 17, 281-300.
47. Smith, J. A. (2004). Reflecting on the development of interpretative phenomenological analysis and its contribution to qualitative research in psychology. Qualitative Research in Psychology, 1, 39-54.

For peer review only

For peer review only - http://bmjopen.bmj.com/site/about/guidelines.xhtml

48. Smith, J. A. (2008). Qualitative Psychology: A Practical Guide to Research Methods. London: Sage.
49. Smith JA, Flowers P, Larkin M (2009). Interpretative phenomenological Analysis: Theory, Method, Research. London: Sage
50. Smith, J. A., Harré, R., & Van Langenhove, L. (1995). Idiography. In J. A. Smith, R. Harré, & L. Van Langenhove, Rethinking Psychology, 56-69. London: Sage.
51. Smith, J. A., & Osborn, M. (2008). Interpretative Phenomenological Analysis. In J. Smith, Qualitative Psychology: A Practical Guide to Research Methods, 53-80. London: Sage.
52. Flowers, P., Smith, J., Sheeran, P. i Beail, N. (1997). Health and romance: Understanding unprotected sex in relationships between gay men. British Journal of Health Psychology, 2, 73-86.
53. Sharp, G. (2019). What is The Russell Group? List of Russell Group Universities. Accessed from: https://www.theuniguide.co.uk/advice/choosing-a-course/what-is-the-russell-group
54. Nurunnabi, M. & Abdelhadi, A. (2018). Student Satisfaction in the Russell Group and Non-Russell Group Universities in UK. Data in Brief. doi: https://doi.org/10.1016/j.dib.2018.11.124
55. Boliver, V. (2016). Exploring Ethnic Inequalities in Admission to Russell Group Universities. Sociology, 50(2), 247–266. https://doi.org/10.1177/0038038515575859
56. Malmqvist, J., Hellberg, K., Möllås, G., Rose, R., & Shevlin, M. (2019). Conducting the Pilot Study: A Neglected Part of the Research Process? Methodological Findings Supporting the Importance of Piloting in Qualitative Research Studies. International Journal of Qualitative Methods. https://doi.org/10.1177/1609406919878341
57. Pietkiewicz, I. & Smith, J.A. (2012) Praktyczny przewodnik interpretacyjnej analizy fenomenologicznej w badaniach jakościowych w psychologii (. Czasopismo Psychologiczne, 18(2), 361-369
58. Shaw, R. (2010). Embedding Reflexivity Within Experiential Qualitative Psychology, Qualitative Research in Psychology, 7: 3, 233 — 243. Doi: 10.1080/14780880802699092
59. Vicary, S., Young, A., & Hicks, S. (2017). A reflective journal as learning process and contribution to quality and validity in interpretative phenomenological analysis. Qualitative Social Work, 16(4), 550–565. https://doi.org/10.1177/1473325016635244
60. Berends, L. & Johnston, J. (2005). Using multiple coders to enhance qualitative analysis: The case of interviews with consumers of drug treatment. Addiction Research & Theory. 13 (4), 373-381, DOI: 10.1080/16066350500102237

For peer review only

61. O'Connor, C., & Joffe, H. (2020). Intercoder Reliability in Qualitative Research: Debates and Practical Guidelines. International Journal of Qualitative Methods. https://doi.org/10.1177/1609406919899220
62. Adamy, E., Zocche, D., Vendruscolo, C., Guedes dos Santos, J., & Almeida, M. (2018). Validation in grounded theory: conversation circles as a methodological strategy. Revista Brasileira de Enfermagem. 71. 3121-3126. Doi: 10.1590/0034-7167-2017-0488.
63. King's College London (2019). King's College London Records and Data Retention Schedule. Accessed from: https://www.kcl.ac.uk/aboutkings/orgstructure/ps/audit/records/retention/rdrs-v7.pdf
64. Alase, A. (2017). The Interpretative Phenomenological Analysis (IPA): A Guide to a Good Qualitative Research Approach. International Journal of Education and Literacy Studies, 5(2), 9-19. doi:http://dx.doi.org/10.7575/aiac.ijels.v.5n.2p.9
65. Creswell, J. W., & Poth, C. N. (2018). Qualitative inquiry & research design: Choosing among five approaches.
66. Austin, Z., & Sutton, J. (2014). Qualitative research: getting started. The Canadian journal of hospital pharmacy, 67(6), 436–440. https://doi.org/10.4212/cjhp.v67i6.1406

For peer review only
